# Two distinct types of remapping in primate cortical area V4

**DOI:** 10.1038/ncomms10402

**Published:** 2016-02-01

**Authors:** Sujaya Neupane, Daniel Guitton, Christopher C. Pack

**Affiliations:** 1 Department of Neurology and Neurosurgery, Montreal Neurological Institute, McGill University, 3801 University Street, #896, Montreal, Quebec, Canada H2A2B4

## Abstract

Visual neurons typically receive information from a limited portion of the retina, and such receptive fields are a key organizing principle for much of visual cortex. At the same time, there is strong evidence that receptive fields transiently shift around the time of saccades. The nature of the shift is controversial: Previous studies have found shifts consistent with a role for perceptual constancy; other studies suggest a role in the allocation of spatial attention. Here we present evidence that both the previously documented functions exist in individual neurons in primate cortical area V4. Remapping associated with perceptual constancy occurs for saccades in all directions, while attentional shifts mainly occur for neurons with receptive fields in the same hemifield as the saccade end point. The latter are relatively sluggish and can be observed even during saccade planning. Overall these results suggest a complex interplay of visual and extraretinal influences during the execution of saccades.

## Abstract (web summary)

Visual receptive fields are known to change positions around the time of a saccade, but the nature of this remapping is unclear. Here Neupane and colleagues show that neurons in area V4 of the visual cortex exhibit two types of remapping, one consistent with a role in maintaining perceptual stability, and a second that seems to reflect shifts of attention.

## Graphical abstract

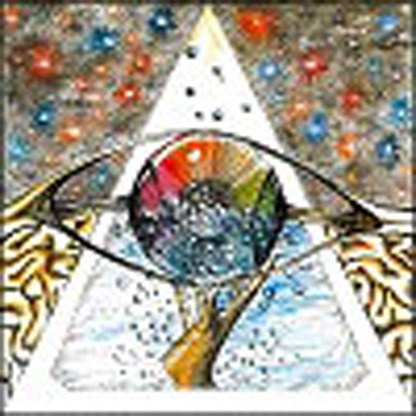

The early visual system contains a retinotopic organization, with nearby neurons encoding information from nearby parts of the retina^1^. Although this organization appears to be established during the early stages of development^2^, it can also change transiently during the execution of saccades. Specifically, numerous studies have reported a change in the position of individual visual receptive fields (RFs) around the time of a saccade. This phenomenon has been termed future field (FF) remapping, because it involves a shift of the RF towards the location it will occupy after the saccade^3^,^4^,^5^,^6^,^7^,^8^,^9^,^10^,^11^. Functionally, FF remapping is thought to provide a linkage between pre- and postsaccadic spatial representations^12^.

In contrast, two studies have shown that RF remapping is directed towards the saccade target (ST)^13^,^14^, irrespective of the future RF position. This ‘saccade target' remapping is more consistent with a role in emphasizing visual information near STs than in achieving visual stability. The two types of remapping might be independent neural properties, or one may be a special case of the other^8^,^14^. At present, there is no way to evaluate these possibilities, because FF and ST remapping have been observed in different studies, which used different paradigms, and usually involved recording from different areas.

Here we report measurements of perisaccadic responses in space and time, based on multi-electrode array recordings in area V4 of macaque monkeys. V4 is known to play an active role in visual perception by integrating visual and oculomotor input, as well as attentional signals^15^,^16^,^17^,^18^,^19^. Our results suggest that FF and ST remapping are distinct features of V4 responses. Indeed we found both types of remapping in the same individual neurons, with ST remapping usually emerging later in the response. Moreover, the two types of remapping depend differently on the saccade vector, with ST remapping being observed primarily for saccades directed towards the hemifield containing the neuron's RF, while FF remapping occurs for saccades in the opposite direction. Overall these results support the existence of both FF and ST remapping, and suggest that the two effects reflect distinct functional influences on V4.

## Results

### Perisaccadic receptive fields have multiple responses

**Figure 1:**
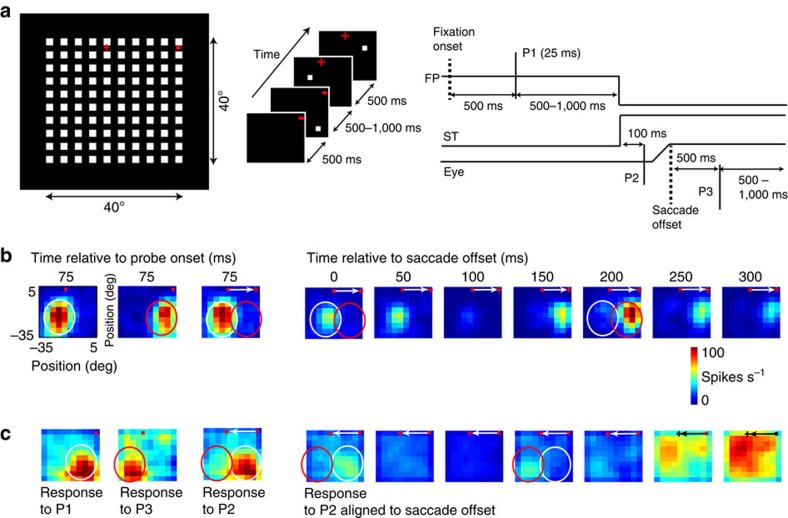
Illustration of experimental paradigm and example neurons. (**a**) (left) All possible visual probe locations (10 × 10 probe grid) spanning 40° of visual space (figure not drawn to scale for clarity). Fixation point (red dot) and saccade target (red ‘+') indicate the leftward direction of the horizontal, visually guided saccade in this example. (middle) Sequence of visual stimuli on an example trial, including the fixation targets and the appearance of the three probes at random locations. (right) Time course of a single trial. (**b**) Receptive field of an example neuron for probes flashed during fixation (left two panels; fixation points indicated by red dot) and those flashed immediately before an ‘away saccade' (defined as a saccade directed horizontally away from the hemifield containing the receptive field) indicated by white arrows (third panel: responses aligned to probe onset; and fourth panel and onwards: responses aligned to saccade offset). (**c**) Receptive field of another example neuron for a ‘towards saccade' (defined as a saccade directed horizontally towards the hemifield contacting the receptive field) (same scheme as in **b**). FP, fixation point; ST, saccade target.

To isolate fixation and perisaccadic responses, we recorded from area V4 while monkeys executed saccadic eye movements towards a continuously lit target. At various times relative to each saccade, we flashed visual probe stimuli sequentially at random locations chosen to span each neuron's RF (Fig. 1a, leftmost panel). Specifically, we flashed a single probe during fixation (P1), another probe just before the initiation of a saccade (P2), and a third probe following the completion of the saccade (P3) (Fig. 1a, middle and right panels). We analysed each neuron's responses to every probe at different times relative to each saccade, in order to obtain the temporal profile of perisaccadic RF shifts.

We studied the responses of a total of 151 neurons that exhibited significant visual responses during fixation. We examined responses to the different probes described above during performance of horizontal saccades both towards (92 neurons) and away (59 neurons) from the visual hemifield containing the RFs of the neurons under study.

Figure 1b shows example data from a single neuron during execution of away saccades. The leftmost panel in the figure represents the responses to probes (P1) flashed while the monkey was fixating near the top, centre of the monitor display (small red dot). Here responses represent averages over a 25 ms window aligned to the onset of the probes, with reddish colours representing strong responses and bluish colours representing weak responses. The white circle represents the limits of the fixation RF, henceforth referred to as the current field (CF).

After a fixation period of 500–1,000 ms, the animal was cued to make an away saccade, to a location indicated by the red dot in the second column of Fig. 1b. Following the execution of the saccade, the neuron responded to probes (P3) in a different location on the screen, indicated by the red circle. The change in RF position after the saccade simply indicates that the neuron's RF is defined in retinal coordinates^20^,^21^. On this basis, FF remapping in this neuron would appear as responses to probes (P2) flashed in the postsaccadic RF before saccade onset.

Responses to P2 probes are shown in the third column of Fig. 1b; here the data are aligned to probe onset, with a peak latency of 75 ms, for probes flashed in the CF. To study perisaccadic RF shifts, we aligned the P2 responses to saccade offset, and these responses are shown in the remaining columns of Fig. 1b. Early responses are confined to the CF (Fig. 1b; fourth column); however, at later time points (Fig. 1b; panels at 100 ms and beyond), the CF response fades and is replaced by a FF response. This is seen most clearly at ∼200 ms after the end of the saccade (Fig. 1b, third column from the right). Note that this response cannot be explained by postsaccadic retinal stimulation, because the probes (P2) disappeared before the initiation of each saccade (see Methods for details and Supplementary Fig. 1 for controls). Thus this neuron clearly exhibited FF remapping for saccades made away from the hemifield containing its RF.

Figure 1c shows the responses of another neuron for towards saccades. As for away saccades (Fig. 1b), responses to the P2 probe initially reflected the location of the CF (Fig. 1c, third column), with a FF response emerging at roughly 150 ms after saccade offset. However, in contrast to the case of away saccades, a third RF emerged somewhat later (near 250 ms after the saccade) (Fig. 1c, rightmost columns). This RF was large and centred in the upper visual field, near the ST.

Thus, for this example neuron, we found that probes flashed immediately before the saccade elicited both types of remapping that have previously been reported in the literature^3^,^14^, with FF remapping being followed by ST remapping. The relative strength of these effects appears to depend on the saccade direction and the time at which the response is measured.

### Remapping for saccades made away from the receptive fields

**Figure 2:**
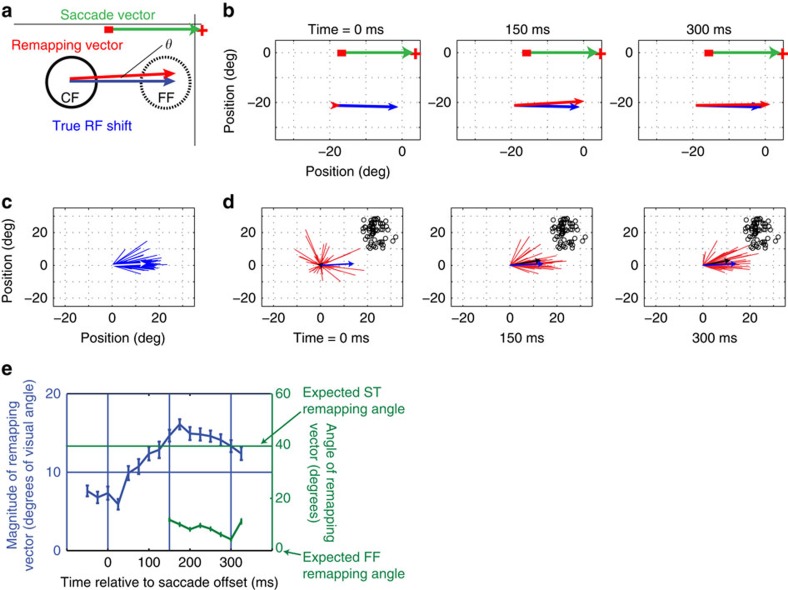
Quantification of receptive field remapping for away saccades. (**a**) A cartoon of the CF (solid black) and the FF (dotted black) of a hypothetical neuron for an away saccade (green arrow). The true receptive field shift is a vector connecting the centres of the CF and FF (blue arrow). Similarly, the actual remapping vector connects the centre of the CF and the centre of the perisaccadic receptive field (red arrow). The magnitude of this vector and its angle of rotation (indicated by *θ*) relative to the true receptive field shift vector (blue arrow) are used to quantify receptive field remapping. (**b**) True RF shift vector (obtained by joining RF centres 75 ms after P1 and P3 onset, respectively) and remapping vector of the example neuron from Fig. 1b at different times relative to the saccade offset. (**c**) True RF shift vectors of the population of 86 neurons. The white arrow indicates the average vector. (**d**) Remapping vectors of the population of cells at different times relative to saccade offset. The black arrows indicate the average vectors. The blue arrow shows the average true RF shift (same as white arrow in (**c**)). Since all the RFs are centred at the origin, the saccade target positions vary (indicated by small black circles). (**e**) Time course of the average magnitude (blue; error bars represent s.e.m. across the population of neurons) and mean direction (green; error bars represent circular s.d.) of the remapping vectors relative to the saccade offset. Vertical lines indicate analysis time points at 150 and 300 ms post saccade, as well as saccade offset at 0 ms. CF, current field; FF, future field.

We quantified the direction and timing of remapping for 86 neurons during away saccades. To distinguish between ST and FF responses, we discarded from the analysis neurons with RF eccentricities below 10° (6/92 neurons). The logic of the analysis is depicted in Fig. 2a, which shows a cartoon of the CF (solid black) and the FF (FF in dotted black) of a hypothetical neuron. The true RF shift is a vector connecting the centres of the CF and FF (blue arrow); this defines the RF shift that would occur if the neuron exhibited FF remapping. Similarly, the actual remapping vector connects the centre of the CF and the centre of the observed perisaccadic RF (red arrow in Fig. 2a). In all cases the RF centres were estimated nonparametrically as the centre of gravity (see Methods) of the responses to all probe locations, as done in previous work^14^.

The geometry shown in Fig. 2a allows us to generate quantitative predictions for the various types of remapping shown in Fig. 1 and in previous work. For FF remapping, on one hand, we should observe a preponderance of actual remapping vectors with angles and magnitudes that are similar to those of the true RF shifts. On the other hand, if there were no remapping, the FF vector angles should be randomly distributed, with lengths near zero. Finally, for ST remapping, we would expect to see a deviation of the actual remapping vector angle away from the FF and towards the ST. Because the neurons in our sample had RFs confined to the lower visual field, an upward displacement of the actual remapping vector is a signature of ST remapping.

Figure 2b shows the actual remapping vector (red) of the example neuron from Fig. 1b superimposed on the true RF shift vector (blue) at different times relative to away saccades. Early in the perisaccadic period (0 ms; Fig. 2b, left panel), the response is still largely confined to the CF (Fig. 1b), so that the remapping vector (red line, not apparent at this scale) has a small magnitude. After the saccade (Fig. 2b, middle and right panels), the responses to the same probes now reflect FF remapping, as the actual remapping vector aligns closely with the true RF shift. Note that although FF remapping emerges after the saccade, the P2 probes were flashed immediately before saccade onset.

Figure 2c shows the distribution of true RF shift vectors across the V4 population, for away saccades. As a population, these vectors are similar to the angle (horizontal) of the saccades executed by the monkey. The magnitude of the true RF shifts is typically close to the actual saccade length (20°), with a small undershoot because of the fact that some RFs extended beyond our probe grid.

Figure 2d shows the actual remapping vectors at various times relative to the saccade end. Here the ST positions, relative to the centre of each CF, are shown as the black circles. Early in the population response (Fig. 2d, left panel) there is no consistent direction of remapping, suggesting that responses were largely confined to the CF (Raleigh non-uniformity test, *P*>0.05, *n*=68). Later in time, the responses to the same P2 probes (Fig. 2d, centre and right panels) were sharply tuned (Raleigh non-uniformity test, *P*<<0.001, *n*=46 and 57, respectively). The means of these angular distributions were not significantly different from 0° (one-sample test; H0: mean slope=0°; *P*>0.05), suggesting that the perisaccadic RFs had shifted in a direction consistent with FF remapping.

We also compared the magnitude of the remapping vector with that of the true RF shift. Across the population these magnitudes differed significantly for the early perisaccadic responses (Fig. 2d, left panel; paired *t*-test; *P*<0.001, *n*=68), but not at later times (Fig. 2d, centre and right panels; paired *t*-test; *P*>0.5, *n*=46 and *n*=57), indicating that neurons first responded in the CFs and subsequently in their FFs.

Figure 2e shows a more detailed time course of perisaccadic remapping for the V4 population, during away saccades. The blue line shows the average magnitude of the remapping vector, measured at 25 ms intervals. A gradual increase in the vector length is apparent with a peak near 150 ms. The green line shows the mean angle of the remapping vector for each subsequent time point, indicating little deviation from horizontal, FF remapping.

The quantification of remapping vectors for individual neurons further confirms that FF remapping was the dominant perisaccadic effect during away saccades. For the V4 population, 82% of the neurons had remapping vector angles that were within 20° of the horizontal axis, while only 7% remapped at an angle >20° (Table 1).

### Receptive-field remapping depends on saccade vector

**Figure 3:**
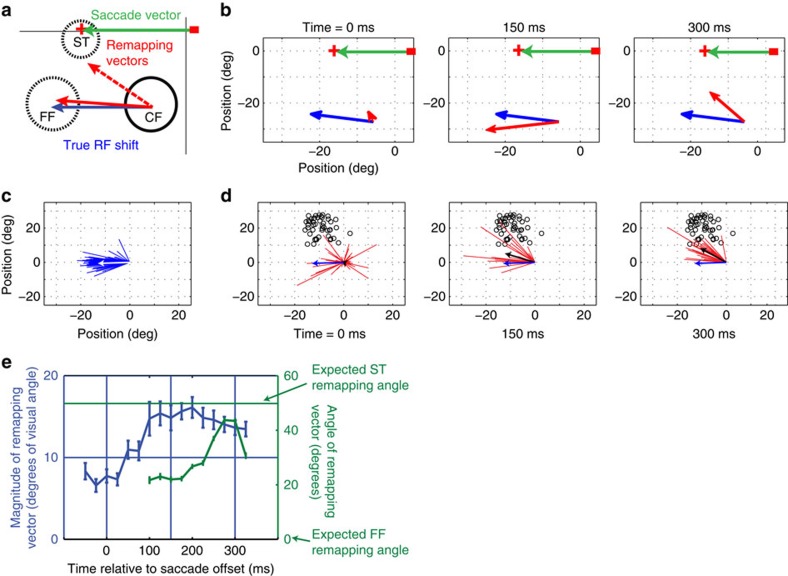
Quantification of receptive field remapping for towards saccades. (**a**) Receptive field geometry, as in Fig. 2a. (**b**) True RF shift vector (obtained by joining RF centres, 75 ms after P1 and P3 onset, respectively) and remapping vector of the example neuron from Fig. 1c at different times relative to the saccade offset. (**c**) True RF shift vectors of the population of 54 neurons. The white arrow indicates the average vector. (**d**) Remapping vectors of the population of cells at different times relative to saccade offset. The black arrows indicate the average vectors. The blue arrow shows the average true RF shift (same as white arrow in (**c**)). Since all the RFs are centred at the origin, saccade target positions vary (indicated by small black circles). (**e**) Time course of average magnitude (blue; error bars represent s.e.m. across the population of neurons) and mean direction (green; error bars represent circular s.d.) of the remapping vectors relative to the saccade offset.

Figure 3b shows the evolution of perisaccadic responses for saccades towards the hemifield of the example neuron whose RF is shown in Fig. 1c. As in Fig. 2b, the response in the early perisaccadic period is largely confined to the CF, as indicated by the small magnitude of the actual remapping vector (Fig. 3b, left panel; red line). After the saccade (150 and 300 ms; Fig. 3b, middle and right panels), the responses to the same probes evolve in time to show a mixture of FF and ST remapping, as the RF centre is displaced away from the FF position towards the ST.

Figure 3c (left panel) shows the distribution of vectors across the V4 population of 54 neurons (5/59 neurons with RF eccentricity <10° were discarded) for the true RF shifts for towards saccades. Again there is a clear mode near zero, which corresponds to the angle of the towards saccades executed by the monkey. As for away saccades, the actual remapping vectors (Fig. 3d, left panel) as a population were initially untuned (Raleigh non-uniformity test, *P*>0.05, *n*=47), indicating no consistent direction of remapping. Later in time, the same distribution was sharply tuned (Fig. 3d, centre and right panels; Raleigh non-uniformity test, *P*<<0.001, *n*=23 and *n*=36, respectively). The means of these angular distributions were not significantly different from 0° (one-sample test; H0: mean slope=0°; *P*>0.05, *n*=23) at 150 ms, but they were significantly different from zero at 300 ms (one-sample test; H0: mean angle=0°; *P*<0.05; circular mean=42°, *n*=36), suggesting that the perisaccadic RFs first shifted to the FF, after which they shifted towards the ST (black circles in each figure panel). The rotation of the mean of the angular distribution at the later time period was significant to a confidence level of 90% (Watson–Williamson two sample test; F_1_, _57_=2.56, *P*=0.122). Moreover, the deviation of the actual remapping vector angle from zero was significantly greater for towards saccades than for away saccades at 300 ms after the saccade (Watson–Williamson two sample test; F_1_, _91_=5.18, *P*=0.03). As for away saccades, the magnitudes of the actual remapping vectors were not significantly different from those of the true RF shifts at 150 ms after the saccade (paired *t*-test; *P*>0.5, *n*=23) or at 300 ms after the saccade (paired *t*-test; *P*>0.5, *n*=36). To verify that these results were not dependent on the luminance configuration of the stimulus, we repeated these experiments with black probes presented on a grey background, and the results were quite similar (Supplementary Fig. 1).

Figure 3e shows the average time course of V4 remapping, for both the magnitude (blue) and the angle (green) of the remapping vector. In contrast to the same analysis for away saccades (Fig. 2e), the mean angle rotates continuously through time, indicating a shift from FF remapping to ST remapping. Overall, 55% of V4 neurons showed a remapping vector consistent with ST remapping for towards saccades (Table 1).

### Future field remapping followed by saccade target remapping

Previous studies have focused on the spatial^14^ or temporal^7^ structure of remapping, but our results suggest that these dimensions are not independent. Indeed, particularly for towards saccades (Figs 1c and 3d), the spatial structure of remapping depends on the time at which it is measured. We therefore examined spatial remapping responses in fine temporal detail.

**Figure 4:**
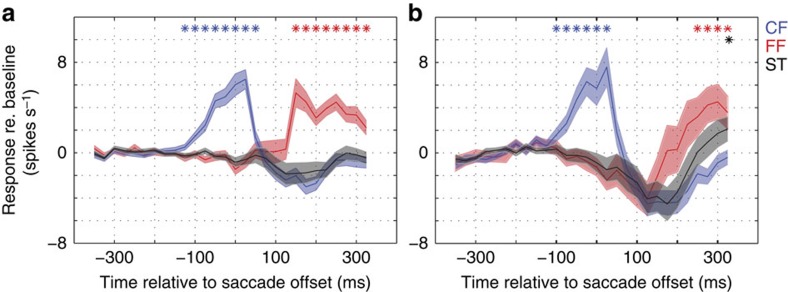
Temporal dynamics of different types of remapping in V4. (**a**) Average responses to P2 probes flashed at the CF (blue), FF (red) and saccade target location (black) for away saccades (*n*=83). Baseline responses before the probe onset were subtracted from the response curves. (**b**) Responses to P2 probes flashed at the CF (blue), FF (red) and saccade target location (black) for towards saccades (*n*=54). Stars indicate responses that were significantly above baseline (one sample *t*-test, *P*<0.05) and shades represent s.e.m. across the respective population of neurons.

We selected three regions of interest (ROIs) on the probe grid corresponding to the CF, the FF and the ST for each neuron. Figure 4a,b show the perisaccadic responses, relative to baseline, for away and towards saccades. In both cases, significant responses were observed at the current (blue lines) and FF (red lines) locations (one sample *t*-test; *P*<0.001, *n*=83). For away saccades, CF responses peaked from −100 to 50 ms relative to the saccade offset, whereas FF responses peaked from 150 to 300 ms after the saccade. For towards saccades (Fig. 4b), a late (300 ms) but significant response was observed for probes flashed near the ST (one sample *t*-test; *P*<0.05, *n*=54) (Fig. 4b, black line). This was not observed for away saccades (Fig. 4b, black line). In addition, there was an inhibitory effect around 125 ms after the saccade, during which responses at all three ROIs were below baseline. Similar plots for example neurons are shown in Supplementary Fig. 2a and b.

In summary, in response to towards saccades, the V4 population shifts its responses from the CF (Fig. 4b, blue line) to the FF (red line) and subsequently to the ST (black line). For away saccades (Fig. 4b), the progression is the same, but the ST response is largely absent. A summary of the frequencies of different kinds of remapping is provided in Table 1.

### Remapping of receptive fields during fixation

One interpretation of these results is that FF and ST remapping reflect different mechanisms that are active around the time of a saccade. FF remapping may be a means of maintaining spatial constancy^3^, while ST remapping may reflect the deployment of attention that occurs with each saccade^14^,^22^,^23^. The latter might occur even in the absence of a saccade, as attention can be shifted covertly, and such shifts have a strong influence on V4 RFs^24^,^25^.

Because the location of the ST was entirely predictable in our visually guided saccade task, the monkey probably directed covert attention towards the expected ST location^22^. This led us to ask if there was any modulation of RF structure in response to P1 probes, which were presented long before (typically 500–1,000 ms) the appearance of the ST (Fig. 1a). To test this idea, we analysed P1 responses during steady fixation. To highlight potential RF shifts, we chose neurons with RFs far from the ST (eccentricities >25°; *N*=45/161).

**Figure 5:**
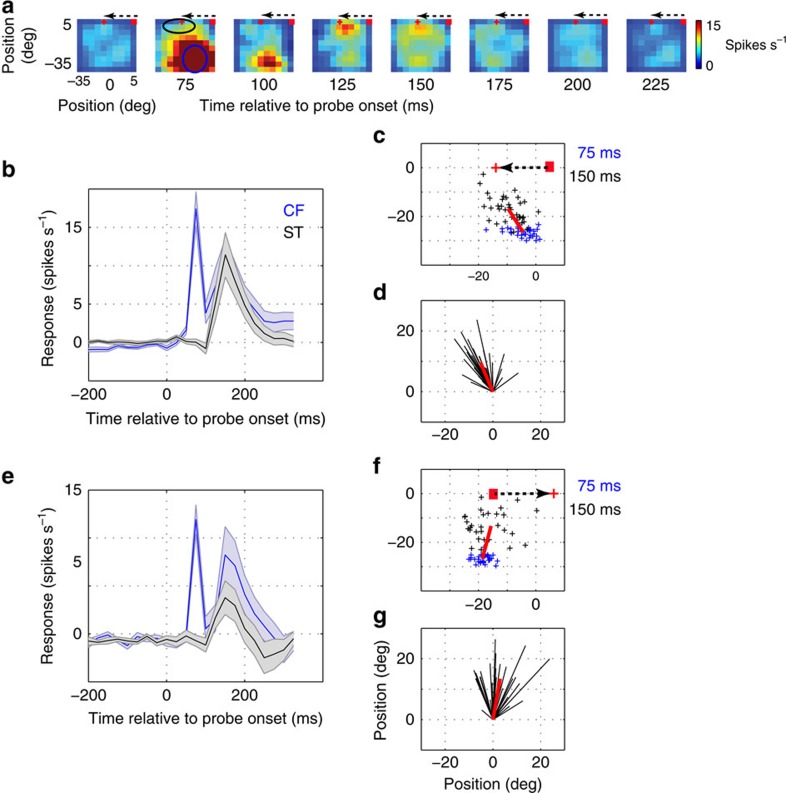
Receptive field remapping during fixation. (**a**) Time course of the receptive field of an example neuron during fixation. The fixation point is indicated by the red dot, and the saccade target is indicated by the red ‘+, which will appear 500–1,000 ms after P1 probe onset. Dotted arrow indicates an impending saccade after the target appearance. (**b**) Average temporal dynamics (*n*=35 neurons) of responses to probes flashed at the CF (blue) (indicated by blue circle in the second panel of Fig. 5a) and those at the saccade target (black) (indicated by black circle in the second panel of Fig. 5a) during fixation before towards saccades. (**c**) RF centres at visual latency of 75 ms (blue ‘+') and those at 150 ms (black ‘+') after probe onset during fixation at the red dot. The red line indicates the vector connecting the average RF locations at the two time points after probe onset. (**d**) The black lines represent the vectors connecting the RFs at 75 ms and RFs at 150 ms after probe onset. RFs at 75 ms are aligned for quantifying the average direction of RF shift. (**e**) Average temporal dynamics (*n*=31 neurons) of responses to probes flashed at the CF (blue) and those at saccade target (black) during fixation before away saccades. (**f**) RF centres at visual latency of 75 ms (blue ‘+') and those at 150 ms (black ‘+') after probe onset during fixation at the indicated red dot. (**g**) Same as **d**. Shades represent s.e.m. across the respective population of neurons.

Figure 5a shows an example neuron's fixation RF, which initially is confined to the lower visual field (second panel from the left). However, at 150 ms after the presentation of the P1 probe, a response is observed near the location of the ST (indicated by the red cross), peaking at 150 ms after probe onset. Note that at this time point, the ST will not appear for another 350–850 ms. This suggests that the monkey's anticipation of the ST influenced the spatiotemporal structure of the neuron's RF.

To characterize the spatiotemporal dynamics of RF modulation during fixation, we chose ROIs at the CF (blue circle), defined by responses at visual latency (75 ms), and near the ST (black oval) (see Methods). Figure 5b shows the average responses in these ROIs when the monkey was anticipating a towards saccade. At the visual latency of 75 ms, significant responses were observed only at the CF. At a later time, however, the population of neurons responded to probes flashed at the future ST; this response was significantly above baseline for 45% (20/45) of the neurons (one sample *t*-test; *P*<0.01, *n*=120 trials). A similar plot for one example neuron is shown in Supplementary Fig. 2d. Figure 5c shows the centre-of-mass of these responses at 150 ms after the probe onset (black crosses), compared to those at 75 ms (blue crosses). The distribution is clearly shifted towards the ST, as indicated by the average RF shift vector (red line). By joining the centres of RFs at the two different time points, we obtained a vector distribution (Fig. 5d) with a mean direction of 117.5^o^ and a non-zero magnitude (one-sample *t*-test, *P*<0.001, *n*=35). This angle was not significantly different from the mean angle of vectors joining the RF centres and the ST (Watson–Williamson two sample test; F_1_, _68_=1.56, *P*=0.21). Thus, even during the fixation period, RFs shifted towards the future ST.

Attention is thought to modulate existing visual responses, without changing stimulus selectivity^26^,^27^. On this basis, we would expect ST responses to be weaker during the anticipation of away saccades, as V4 RFs are confined to the contralateral visual hemifield^20^. We therefore examined responses to P1 probes in the away saccade condition (Fig. 5e). ST responses were weaker than in the towards saccade condition. A similar plot for one example neuron is shown in Supplementary Fig. 2c. Moreover, the distribution of RF shifts (Fig. 5f,g) had a mean direction (82.3^o^) that was significantly different from that of the ST (Watson–Williamson two sample test; F_1_, _64_=22.8, *P*<<0.0001).

These results are consistent with the idea that ST remapping reflects attentional modulation of V4 RFs, which are insensitive to stimuli in the opposite hemifield. This highlights a clear difference with FF remapping, for which RF shifts are quite strong during away saccades (Figs 2 and 3; see also ref. 28). Of course, since we did not explicitly measure attention, the remapping effect observed during fixation could also be a result of motor planning or changes in alertness, rather than attention. Irrespective of the underlying mechanism, the observed ST remapping occurs even in the absence of an imminent saccade. Moreover, the strength of the fixation remapping response depended significantly on the direction of the impending saccade

### LFP remapping

Previous work has demonstrated a retinotopic organization of the local field potentials (LFPs) in area V4 (ref. 19). Although the LFP retinotopy correlates well with that of the spiking activity during fixation^21^, it is not known whether a similar correspondence is observed during saccades.

**Figure 6:**
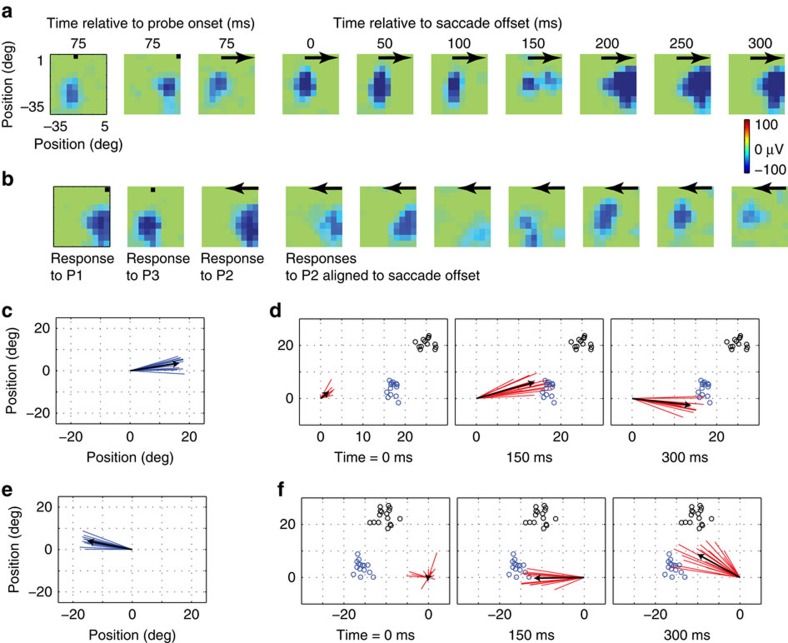
Remapping of LFP receptive fields. (**a**) LFP receptive field of an example electrode for probes flashed during fixation (left two panels; fixation points indicated by black dot) and those flashed immediately before an away saccade, indicated by black arrows (third panel: responses aligned to probe onset; fourth panel and onwards: responses aligned to saccade offset). (**b**) LFP receptive field of an example electrode for towards saccades. (**c**) True LFP RF shift vectors of the population of 14 electrode sites with eccentricity >20°. (**d**) Remapping vectors of the same population at different times relative to saccade offset. The black arrows indicate average vectors. Since all the RFs are centred at the origin, saccade target positions vary (indicated by small black circles). Each vector's expected FF location (based on P3 RF) is indicated by small blue circle. (**e**) and (**f**) show the results from the same analysis as **c** and **d** for towards saccade. LFP, local field potential.

Figure 6a shows the LFP RF of an example electrode from one monkey (similar results from the second monkey are shown in Supplementary Fig. 3). Here blue colours indicate strong modulations of the LFP signal. The format of this figure is the same as Fig. 1, with leftmost columns showing RFs obtained during fixation and the subsequent columns showing the temporal evolution of responses to P2 probes, flashed immediately before saccade onset. For away saccades, there is a clear signature of FF remapping that arises roughly 200 ms after the saccade (Fig. 6a, rightmost columns). For towards saccades, there is again a late response that appears to reflect a mixture of FF and ST remapping (Fig. 6b). Thus the pattern of perisaccadic LFP responses appears to be similar to that observed for spikes.

We quantified this LFP remapping, using the same centre-of-mass analysis that we applied to the spiking responses. Early in the perisaccadic population response (Fig. 6d, first panel) there is no consistent direction of remapping, suggesting that LFP responses were largely confined to the CF (Raleigh non-uniformity test, *P*>0.05, *n*=14). Later in time, the actual remapping vectors in response to the same P2 probes (Fig. 6d, centre and right panels) were sharply tuned (Raleigh non-uniformity test, *P*<<0.001, *n*=14) and not significantly different from 0^o^ (one-sample test; H0: mean slope=0^o^; *P*>0.05, *n*=14), suggesting that the perisaccadic LFP RFs had shifted in a direction consistent with FF remapping.

Figure 6e shows the distribution of true RF shifts obtained from peripheral LFP RFs for towards saccades. As for the spikes, the early population response was untuned (Raleigh non-uniformity test, *P*>0.05, *n*=16; Fig. 6f, first panel), while the later response (Fig. 6f, centre and right panels) was sharply tuned (Raleigh non-uniformity test, *P*<<0.001, *n*=16), with an angle that was significantly different from zero at 300 ms (one-sample test; H0: mean angle=0°; *P*< .05, *n*=16; circular mean=37°). This suggests that the perisaccadic LFP RFs first shifted to the FF and subsequently towards the ST.

## Discussion

We have shown that visual RFs in area V4 remap around the time of saccades, with some RFs shifting towards the ST (42% for towards and 5% for away saccades; see Table 1), others towards the FF (26% for towards and 74% for away saccades), and others showing both types of behaviours at different latencies (17% for towards and 5% for away saccades). These results hold for single neurons and for LFPs. The prevalence of each type of remapping depends strongly on experimental conditions, with FF remapping being largely independent of saccade direction. ST remapping, in contrast, is observed mainly for saccades made into the visual hemifield containing the RFs under study; it can also be observed during fixation, if the subject is anticipating a saccade in the appropriate direction.

In principle, the differences in remapping responses observed in previous studies could have arisen from differences in the paradigms or from differences in recording sites. FF remapping has generally been reported in studies that used coarse spatial sampling, and it has been argued that a more thorough probing of space reveals that remapping is directed mostly or exclusively towards the ST in the frontal eye field (FEF)^29^ and in V4 (ref. 13). At the same time, these studies used a rather coarse sampling of time, which may have obscured the FF remapping that often precedes ST remapping (Fig. 4). This distinction becomes particularly difficult to assess in neurons with foveal RFs, for which the FF and ST locations overlap. We have largely avoided this issue by focusing on neurons with eccentric RFs.

A second important paradigm difference is the direction of the saccade. Our results suggest that robust FF remapping is observed most clearly in V4 when the saccade is directed away from the hemisphere containing the RF (Fig. 2), which has seldom been examined in previous studies of ST remapping (refs 13, 14). This difference between our approach and previous ones may account for the relatively high frequency (82%) of remapping neurons we found in V4; when saccade direction has been varied in previous work, the fraction of remapping cells has also been quite high (86% of neurons in LIP (ref. 28)). Thus our work highlights the fact that a thorough examination of different saccade vectors, different spatial probe positions, and different perisaccadic time epochs is necessary to fully characterize remapping responses.

A final issue with different paradigms is the timing of the visual stimuli used. The only other study of remapping in V4 (ref. 13) used visual probes that were static throughout each trial, rather than being flashed briefly. This approach precludes a detailed temporal analysis, particularly for long-latency responses of the kind reported here. The time of probe presentation relative to saccade onset is also of some importance: Previous work in FEF has revealed both ‘predictive' responses^3^,^4^,^5^, which occur shortly after saccade offset and ‘memory responses'^30^, which occur later in time. Our FF remapping responses are of the latter variety. While this could reflect a difference between V4 and FEF, it could also be due to the fact that our probes were presented at a time point close to the onset of the saccade. We did this in order to maximize the strength of the remapping response^7^, but it may have obscured potential predictive remapping responses in V4.

Regarding differences in brain regions, the original discovery of FF remapping in parietal cortex^3^ was followed by similar findings in other parts of the oculomotor system, including FEF (ref. 5) and superior colliculus^4^,^9^,^31^. In the visual system, FF remapping appears to be more common at higher levels of the cortical hierarchy^6^. One might expect a prominent role for remapping in the dorsal pathway that culminates in parietal regions concerned with spatial representations, but recent work shows a lack of RF shifts in a key node along this pathway, the middle temporal area^32^. At the same time, our results demonstrate strong FF remapping in V4, which is typically considered to be an important part of the ventral pathway for shape recognition^33^.

The source of ST remapping is likely to be the FEF, which appears to play a role in top-down modulation of V4 responses^34^,^35^,^36^,^37^. Consistent with our observations on towards saccades, this influence is known to be ipsilateral^38^, limiting attentional influences to the contralateral visual field (Figs 3 and 4). At the same time, blocking the corollary discharge pathway from the brainstem to FEF only partially eliminates behavioural correlates of remapping^39^. Since V4 receives input from other areas that exhibit remapping, such as LIP^40^, it could be the case that ST remapping and FF remapping are two different functionalities involving different neuronal circuitries.

Previous psychophysical studies have reported a dramatic distortion of the perception of visual space around the time of a saccade^41^,^42^,^43^. A prominent component of this distortion is called perisaccadic compression: Visual targets flashed around the time of a saccade are typically perceived to be closer to the ST than they really are^42^. The neural basis of such distortions has often been hypothesized to be in the dorsal visual pathway^44^. However, there is a clear distinction between FF remapping, which follows a fixed vector, and perceptual compression, which entails shifts in opposite directions for stimuli placed on either side of the ST^43^,^45^. Alternatively the perceptual effect has been related to the ST remapping that is observed in FEF^14^. This latter conclusion is heavily model-dependent, as a RF shift directed towards the ST could equally well result in a perceived expansion of visual space away from the ST. In any case, to the extent that the pattern of remapping found in our data affect perisaccadic perception, we would expect to find clear differences in localization for probes presented ipsilateral and contralateral to the fixation point, as the latter will be far less influenced by ST remapping (Fig. 2). Although such probes have occasionally been used in previous studies^42^,^45^,^46^, the existing data are quite sparse; more experiments would be necessary to clarify this issue.

Remapping was previously studied in dorsal areas like LIP, VIP, V3A and other cortical (FEF) and subcortical structures (SC), which lack selectivity for specific stimulus features^47^. In contrast, neurons in V4 are strongly selective for features such as orientation, colour and curvature^48^,^49^,^50^. Although we have not varied the shape or colour of the probe stimuli in this study, we suspect that remapping is largely independent of these features, given that most neurons (up to 82%) showed remapping with our standard stimuli. However, there are theoretical reasons to think that feature-specific remapping is gated by attention^51^,^52^,^53^, and this could be studied with future experiments in V4. Indeed the occurrence of RF remapping in V4 introduces a possible link between pathways selective for object position and those selective for object features such as shape, as suggested also by a recent study in LIP^54^.

## Methods

### Electrophysiological recordings

The recording methods have been described previously^19^,^21^. Briefly, a sterile surgical procedure was carried out to implant a headpost and a chronic 10 × 10 microelectrode (Utah array; Blackrock Microsystems) array in area V4 of two monkeys (*Macaca fascicularis*; monkey N: female, 8 years old and monkey C: male, 10 years old). After recovering from the surgery, monkeys were chair trained (Crist Instruments) and rewarded to make visually guided saccades. All aspects of the experiments were approved by the Animal Care Committee of the Montreal Neurological Institute and were conducted in compliance with regulations established by the Canadian Council of Animal Care.

### Signal acquisition and pre-processing

Wideband signals were recorded using a standard data acquisition system (Plexon Multichannel Acquisition Processor System). Custom modification of the preamplifier and preliminary signal processing were carried out as described previously^19^. In short, spikes were sorted offline for each recording by first band-pass filtering the raw signal between 500 and 4000 Hz and then using a modified ‘wave-clus' algorithm^55^. LFP signals were cleaned by removing action potential waveforms from the wideband signal using a Bayesian spike removal algorithm^56^; the despiked signal was then band-pass filtered (0.2–150 Hz) and downsampled to 500 Hz.

All the data reported here were recorded on non-consecutive days, with at least a week between sessions. Thus it is unlikely that the same neurons appeared in different data sets. In order to further avoid duplication of neurons on neighbouring electrodes or on multiple days, we displayed a sequence of 100 images, repeated 10 times, while the animal maintained fixation. We computed pairwise correlations of the response patterns and inter-spike interval histograms. Only one neuron was rejected on this basis. As an additional check for repeated inclusion of identical units, we implemented the method of Dickey *et al.*^57^; we did not find any additional instances of units being counted more than once. We used the earliest recording session as sample data to obtain true positive and true negative scores. As in Dickey *et al.*, the stability score (*S*) was a quadratic discriminant function as follows:


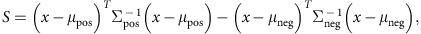


where 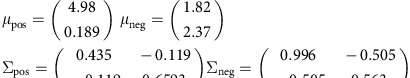


A pair of neurons was counted as the same if *S*<*T*, where *T* is a threshold defined by the mean of the true positive scores plus 3 s.d. (*T*=4.04).

### Experimental paradigm

Visual stimuli were back-projected on a semi-transparent screen by a cathode ray tube video projector, with a refresh rate of 75 Hz. The screen covered an area of 80 × 50° of visual angle at a viewing distance of 78 cm. All visual stimuli were white square probes (luminance=22.5 cd m^−2^) presented for 25 ms on a dark background (luminance <0.01 cd m^−2^).

Each trial started with the animal fixating a red dot 0.5° in diameter. If fixation deviated from the target by more than 2.5°, the trial was aborted. After 500 ms of fixation, a visual probe (P1) was flashed at a randomly chosen location from 100 different locations arranged in a 10 × 10 grid in the lower left quadrant of the visual field (Fig. 1a). The size and location of the grid were chosen such that it covered the retinal eccentricity of all the simultaneously recorded neurons, which was roughly 40° in the lower left hemifield. Each probe was square in shape, 2° in width and was spaced at 4° (centre-to-centre) from its neighbours in the horizontal and vertical dimensions. After a variable delay of 500–1,000 ms, the fixation point jumped to a new target. A second probe (P2) was flashed 100 ms after the appearance of the ST but before saccade initiation. To ensure that P2 flashes were completely off, luminance decay on the projector screen was measured with a photodiode. Luminance decreased by 99% 6 ms after the probe offset; any trial in which the saccade started <10 ms after the probe offset was discarded.

The location of the P2 probe was chosen randomly on each trial from the same 10 × 10 probe grid (Fig. 1a). The monkey was required to make a visually guided saccade to the target and fixate there for another 500 ms. A third probe (P3) was then flashed while the monkey was required to fixate for another variable time of 500–1,000 ms. The trial ended with a liquid reward to the monkey. Trials were repeated such that there were at least 10–15 trials per probe location. Simultaneous, multiple probe flashes were avoided because remapping has been shown to be reduced or even abolished by distracters^9^,^10^.

Eye position was monitored at 1,000 Hz using an infrared eye tracker (Eyelink; SR Research). The liquid reward was only given if the monkey successfully made visually guided saccade followed by additional fixation. Eye movements were processed offline to discard trials that contained blinks, double step or catch-up saccades.

### Eye movements

Saccade onset was defined as the time when the eye trace left the fixation window and crossed a velocity threshold of 80^o^ s^−1^. Saccade offset was the time when eye trace decreased its velocity below 80^o^ s^−1^. If, on any trial, the vertical eye trace was not within the saccade window or the fixation window (±2.5°), it was discarded.

### Estimation of firing rates

To have an unbiased sample, we initially included every neuron that had a discernible waveform. Out of 161 neurons, 10 had firing rates below 2 spikes s^−1^ and were excluded from the analysis, leaving 151 neurons for the analyses described in the Results section.

The peristimulus firing rate of each neuron was estimated as a time histogram by binning spike times into 25 ms bins, starting 350 ms before and ending 350 ms after the probe onset. Similarly, perisaccadic firing rate was estimated by using spike times 350 ms before and after the saccade offset.

For Peri-Stimulus Time Histogram (PSTH) analyses (Figs 4 and 5), the baseline response of every neuron was computed by averaging activity during the initial fixation period, between 275 and 75 ms before P1 probe onset. This was subtracted from the firing rates to obtain the stimulus-modulated responses.

To categorize neurons as FF only, ST only, both or neither, we compared data across the 150 and 300 ms time points used in previous analyses. Given that saccades were made horizontally, we classified neurons as exhibiting FF remapping if their remapping vector angle was within 20^o^ of horizontal. Neurons with vectors deviated more than 20^o^ upward were classified as exhibiting ST remapping. If a neuron exhibited FF remapping at 150 ms and ST remapping at 300 ms, we classified it as an ‘FF and ST' neuron. If a neuron exhibited FF remapping at one or both time points, but exhibited no ST remapping, it was classified as an ‘FF neuron'. ‘ST remapping' were classified analogously. ‘Neither' neurons were those that showed no statistically significant responses to probes outside their classical RFs.

### RF centre estimation

The centre of each RF was estimated as the centre of mass of activity over the probe grid at each time point. This method has been used previously to measure perisaccadic RF centres^14^. To reduce noise we included only statistically significant responses, as defined above. The thresholded RF map was then smoothed with Gaussian filter of size 3 × 3 and s.d. 0.8. The smooth RF map was then interpolated in two dimensions to obtain a resolution of 1 × 1°. After that, each pixel location of the map was weighted by its corresponding firing rate. The weighted probe locations were summed and divided by the total firing rate to obtain a centre of gravity of RF response in Cartesian coordinates, as follows:


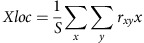



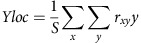


where 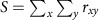.

To obtain the temporal dynamics of neuronal responses (Figs 4 and 5b,e, and Supplementary Fig. S1a and b) to probes at various locations, ROIs were defined based on the estimated RF centres. ROIs were 3 × 3 probe locations in size and centred on the CF and FF; responses to all stimuli within an ROI were averaged to obtain the corresponding CF or FF response. To avoid overlap with the FF ROI, we defined the ST ROI as containing 4 × 2 probe locations centred at the ST. Each response was compared to its corresponding pre-stimulus or pre-saccadic baseline to test for significance (one sample *t*-test, *P*=0.05).

### LFP analysis

As mentioned in our previous paper 21, LFP responses in V4 can be decomposed into a global component, which is nearly constant across recording sites, and a retinotopic component, which is of interest here. The LFP analysis is further complicated by the fact that the global component differs significantly across monkeys. We therefore eliminated the global response by subtracting the P1 response from the P2 response (Supplementary Fig. 3a and b, top panels). The remaining retinotopic signal could then be evaluated for possible remapping effects. We then computed the relationship between probe stimuli and the negative component of the broadband LFP signals (0.2–150 Hz). Significant LFP responses were obtained at every functional electrode, even when there were no spikes recorded at the electrode. To quantify LFP receptive field remapping, we used our centre-of-mass approach to obtain remapping vectors by connecting the centres of the P1 RFs (at visual latency) and those of the P2 RFs at different latencies after P2 onset.

## Additional information

**How to cite this article:** Neupane, S. *et al.* Two distinct types of remapping in primate cortical area V4. *Nat. Commun.* 7:10402 doi: 10.1038/ncomms10402 (2016).

## Supplementary Material

Supplementary Information Supplementary Figures 1-3

## Figures and Tables

**Table 1:** Proportion of cells showing different types of remapping.

**Types of remapping/saccade direction**	**Away**		**Towards**	
	**Time relative to saccade offset**		**Time relative to saccade offset**	
	**150 ms (*****n*****=46)**	**300 ms (*****n*****=57)**	**150 ms (*****n=*****23)**	**300 ms (*****n*****=36)**
FF remapping	82%	66%	52%	27%
ST remapping	7%	8%	34%	55%
	(*n*=68)		(*n*=42)	
FF only	74%		26%	
ST only	5%		42%	
FF and ST	5%		17%	
Neither	16%		17%	

FF, future field; ST, saccade target.
