# Supplementary material for: Two distinct types of remapping in primate cortical area V4
Source: Nat Commun. 2016 Feb 1;7:10402. doi: 10.1038/ncomms10402 (PMC4740356; doi:10.1038/ncomms10402)
Supplement: Supplementary Information — Supplementary Figures 1-3 [file ncomms10402-s1.pdf]

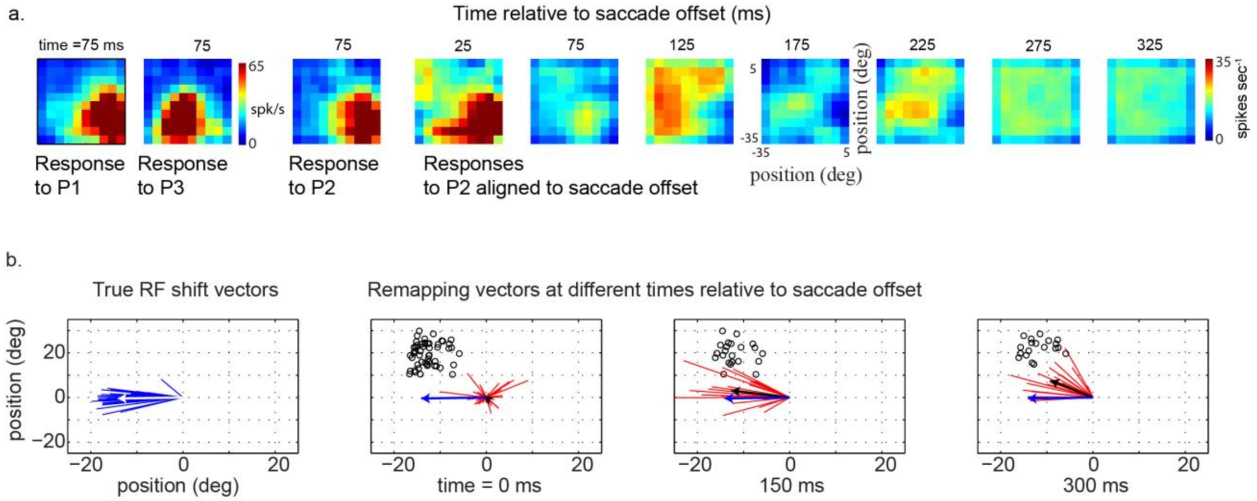

Supplementary Figure 1 | Receptive field remapping with dark probes on gray background: We verified that the results were not due to persistence of the bright probe stimuli on the dark background, by collecting data from toward saccade experiments with dark stimuli on gray background. This configuration yields results similar to those shown in Figure 3. (a) Receptive field of an example neuron for dark probes flashed during fixation (left two panels; fixation points indicated by red dot) and those flashed immediately prior to a '*toward* saccade' indicated by black arrows (third panel: responses aligned to probe onset; fourth panel and onwards: responses aligned to saccade offset) (b) Leftmost panel: True RF shift vectors (obtained by joining RF centers, 75ms after P1 and P3 onset respectively) of the population of 57 neurons. The white arrow indicates the average vector. Second panel onwards: Remapping vectors of the population of cells at different times relative to saccade offset. The black arrows indicate average vectors. Since all the RFs are centered at the origin, saccade target positions vary (indicated by small black circles). For the population of 57 cells tested with dark probes, the difference in the magnitude of the true RF shifts and the magnitude of the remapping vectors in the early perisaccadic response was significantly greater than zero (paired t-test; $p<.01$, $n=52$), whereas those at 150 ms and 300 ms after the saccade were not different from 0 (paired t-test; $p>.5$, $n=22$ and $n=19$). The angle of the remapping vectors was significantly tuned (Raleigh non-uniformity test, $p<<.001$) at 150 ms and 300 ms. The mean angle at 150 ms was 13° (not significantly different from 0; one-sample test: H0=0, $p>.5$, $n=22$), and that at 300 ms was 42° (significantly different from 0; one-sample test: H0=0, $p<.05$, $n=19$). The rotation of the mean of the angular distribution at the later time period was significant (Watson-Williamson two sample test; $F_{1, 39} = 12.61$, $p = .001$).

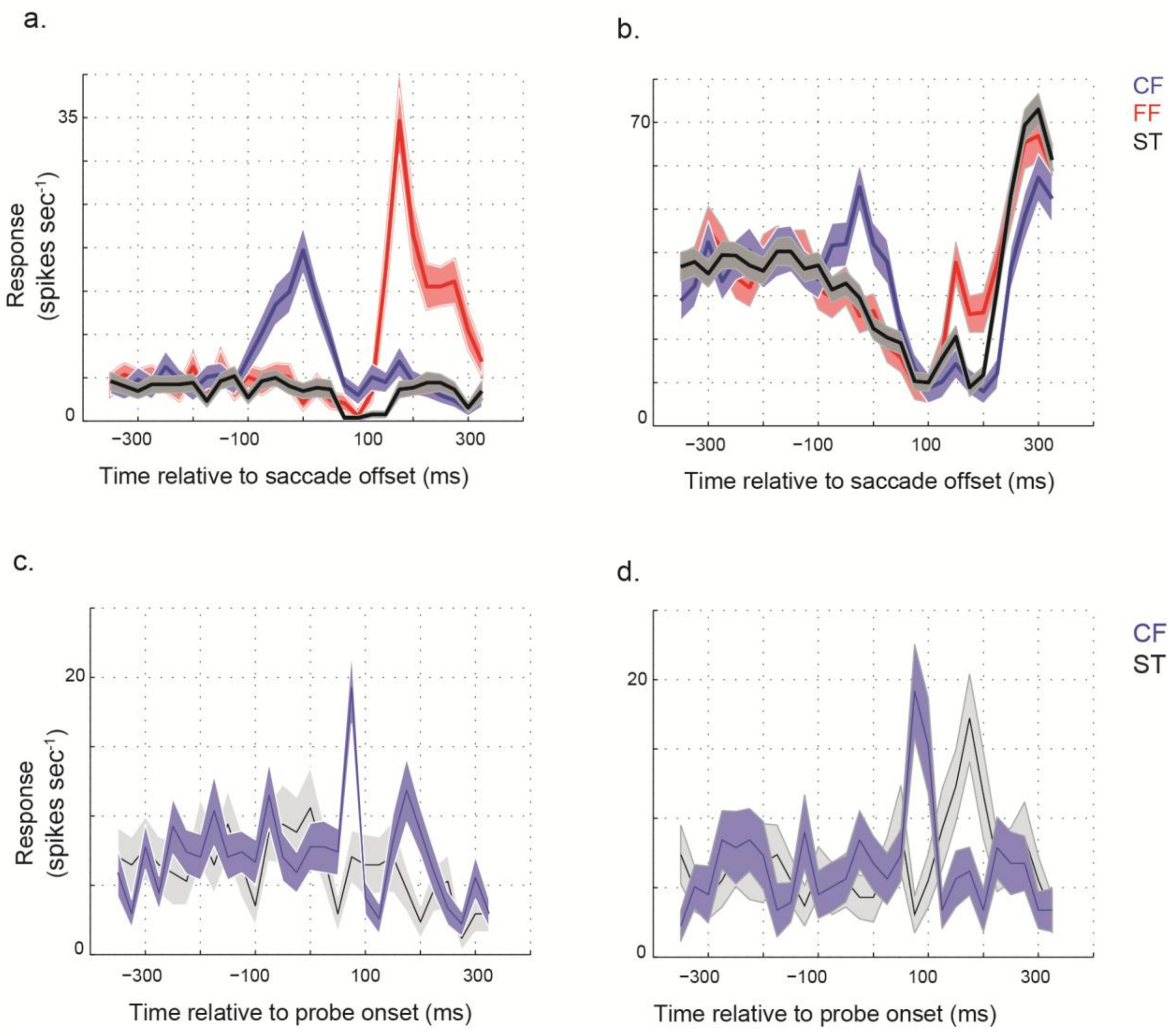

Supplementary Figure 2 | Temporal dynamics of example neurons (a) Peri-saccadic response of an example neuron to P2 probes flashed at the current field (blue), future field (red) and saccade target location (black) for away saccades. (b) Same as (a) for toward saccades. (c) Response of an example neuron to probes flashed at current field (blue) and saccade target (gray) for imminent (away) saccade target during fixation. (d) Same as (c) for imminent toward saccade.For each figure, the shading represents standard error of the mean across all the trials pooled together (120) when probes were flashed in the respective ROIs (CF, FF or ST).

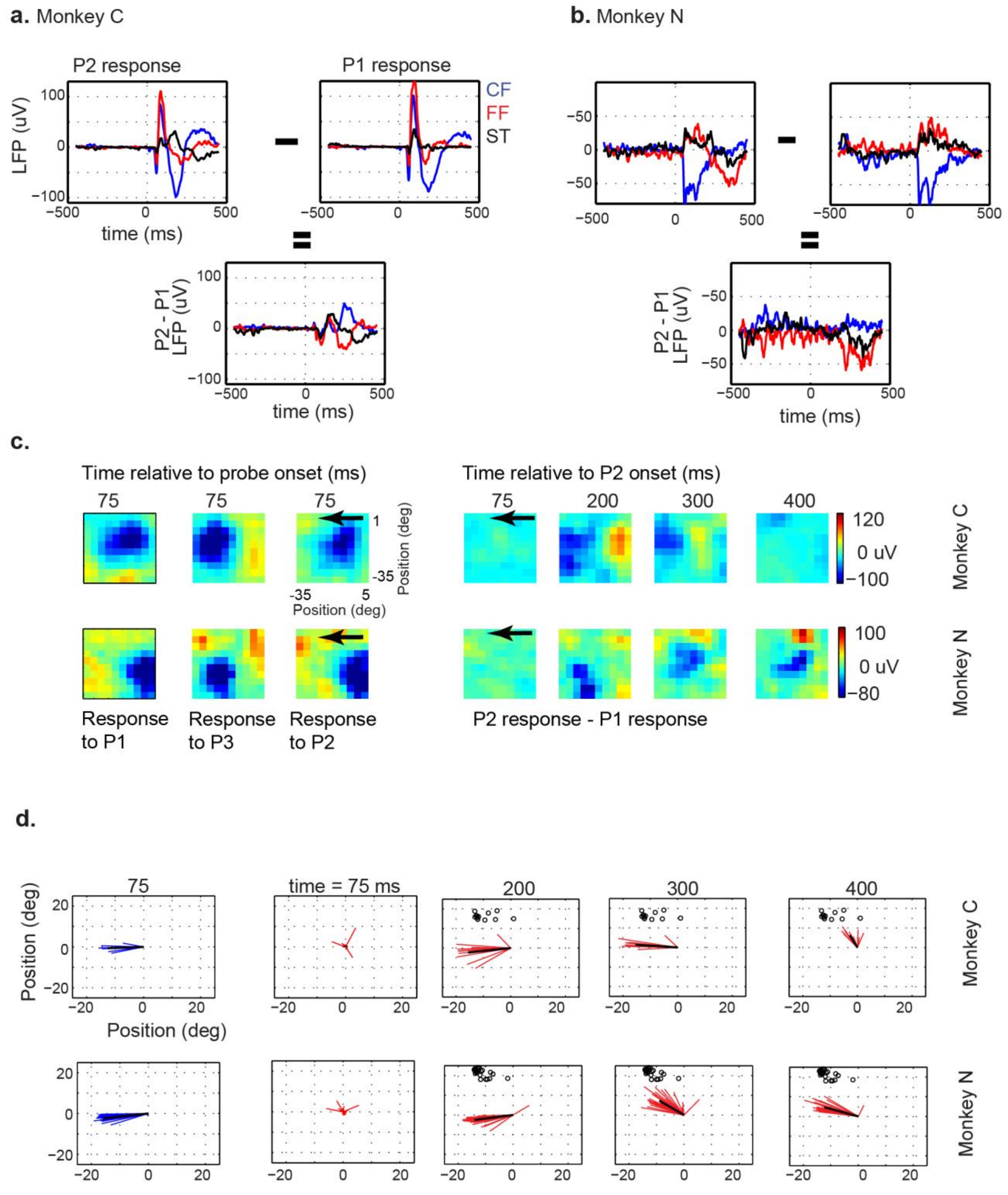

Supplementary Figure 3 | Removing global response from LFP signal to obtain retinotopic component (toward saccade). (a) Temporal dynamics of LFP response at an example recording site from monkey C (top left panel: P2 response; top right panel: P1 response) for probes flashed at the current field (blue), future field (red) and saccade target (black). The bottom panel shows the difference of P2 and P1 responses. This operation yielded significant remapping responses at

the FF and ST for both the monkeys at comparable latencies (one sample t-test, $p < .0001$, n=120 trials). (b) Same as in (a) for monkey N. (c) LFP receptive field of the same two example recording sites for probes flashed during fixation (left two columns) and those flashed immediately prior to the saccade (third column). Fourth column and onwards show receptive fields at different times relative to probe onset obtained after P2-P1 subtraction. (d) Left column: True LFP RF shift vectors (blue lines) of a population of electrodes (top row for monkey C; bottom row for monkey N). Second column and onwards: Remapping vectors at different times relative to P2 onset (red lines) of the same populations with saccade target locations indicated by small black circles. For both the monkeys, LFP receptive fields first remapped to the future field and later toward the saccade target measured by the angular distribution of remapping vectors (Monkey N: mean angle at 200ms (-4°) after P2 ≠ mean angle at 400ms (22.4^{o}) after P2, $F_{1,60} = 36.21$, $p << .0001$; Monkey C: mean angle at 200ms (3°) after P2 ≠ mean angle at 400ms (72.4^{o}) after P2, $F_{1,24} = 40.50$, $p << .0001$;). Compared to the true RF shift vectors (first column), the angular distribution of remapping vectors at early times (200ms) was not different (Monkey N: Watson-Williamson two sample test, $F_{1,60} = 0.005$, p=0.94; Monkey C: $F_{1,24} = 0.712$, p=0.407) but was significantly different at longer latency (400ms) (Monkey N: $F_{1,60} = 67.68$, $p << .0001$; Monkey C: $F_{1,24} = 33.34$, $p << .0001$).
